# Ueber die Wirkung des neuen Tuberkulins TR auf Gewebe und Tuberkelbacillen

**Published:** 1899-09

**Authors:** 


**Ueber die Wirkung des neuen Tuberkulins TR auf Gewebe und Tuberkelbacillen.** Von Dr. H. STROEBE. Pp. 114. Jena: Gustav Fischer. 1898.

Although the practical results of this last tuberculin have been hitherto almost *nil*, we welcome the record of Dr. Stroebe's careful investigations on its true properties and reactions. If any progress is to be made in applying this and similar remedies to the cure of human and animal tuberculosis, or in preventing its spread, the result can only be obtained by an exact study of its effects.

We find here details of researches on healthy and tubercular animals, and on the degree of immunity conferred by the new remedy; and if the results, both in the way of cure or in the production of immunity, are directly opposed to those of Koch, the sooner a dangerous treatment is abolished the better. The writer, we may remark, obtained in no case a complete cure of experimental tuberculosis in guinea-pigs by the use of the tuberculin, and he seems likewise to have failed to produce immunity by its means. He gives also a review of some of the chief investigations which have lately appeared, and of the controversy arising from them.

